# Automatic skin flash optimization in breast and chestwall VMAT with static angle modulated ports: Effect of HU and flash margin size on plan quality and robustness

**DOI:** 10.1002/acm2.70036

**Published:** 2025-02-21

**Authors:** Emily Hubley, Brandon Koger, Taoran Li, Michael Salerno, Ryan M. Scheuermann, Lei Dong, Boon‐Keng Kevin Teo

**Affiliations:** ^1^ Department of Radiation Oncology Perelman School of Medicine University of Pennsylvania Philadelphia Pennsylvania USA; ^2^ Medical Affairs Varian Medical Systems

**Keywords:** breast, motion management, skin flash, treatment planning, VMAT

## Abstract

Skin flash is typically added to breast and chestwall plans to ensure robust target coverage in the presence of respiratory motion, anatomic changes, and small setup uncertainties. Adding skin flash in volumetric modulated arc therapy (VMAT) plans is an iterative and manual process. RapidArc dynamic (RAD) is a new solution that integrates a dynamic collimator and static‐gantry angle modulated ports directly into arc delivery. The automatic skin flash tool (ASF) allows users to automatically add skin flash directly within the optimizer. The user must select the thickness and Hounsfield Units (HUs) of the flash region, but the optimal values are not currently known. For 13 left‐ and right‐sided breast and chestwall patients, RAD plans were created with no skin flash and with ASF with thickness of 5 to 20 mm and HU of −500 to 0 HU. To assess plan quality, DVH metrics for planning target volume (PTV), heart, ipsilateral lung, contralateral lung, and contralateral breast were recorded. To assess plan robustness, the isocenter was shifted 5 mm, moving the target 5 mm anteriorly into the flash region. The changes in clinical target volume (CTV) D95% and D99% were recorded. A paired *t*‐test was used to determine if changes in plan quality or robustness were statistically significant (*p* ≤ 0.05). The addition of ≥ 7 mm of skin flash resulted in robust plans. Varying the HU did not affect robustness. Increasing the skin flash beyond 10 mm increased PTV V105%. This increase was much larger in the 0 HU plans than in the −350 HU plans. We therefore recommend using −350 HU and 7–10 mm of skin flash for anticipated inter‐ and intra‐fraction motion of 5 mm.

## 1 INTRODUCTION

Adjuvant radiation therapy (RT) is essential in the management of breast cancer and has been demonstrated to reduce both recurrence and breast cancer mortality after breast conserving surgery^1^ and in the node‐positive post‐mastectomy setting.^2^ In a thorough review of the literature, Michalski et al. report mean intrafraction breast motion to be 1–2 mm, but motion can be as large as 25 mm.^3^ For interfraction random errors, they report means of 1.9–3.2 mm and a maximum of 23 mm. Conventionally, breast or chestwall RT is delivered using tangential treatment fields with generous skin flash to be robust to these inter‐ and intrafraction motions, as well as breast swelling and other patient anatomic changes.

Volumetric modulated arc therapy (VMAT) has been demonstrated to reduce dose to organs at risk (OAR) and improve target dose homogeneity compared to 3D conformal RT and static‐gantry intensity modulated radiation therapy (IMRT) in some breast cases.^4^, ^5^, ^6^ One difficulty with VMAT breast planning is that many treatment planning systems (TPS) lack the ability to add flash during VMAT optimization. Breast or chestwall VMAT plans have been demonstrated to be less robust to inter and intrafraction motion than tangential treatment plans.^7^, ^8^

A number of studies report using a “virtual bolus” or “pseudo flash” in the Eclipse Treatment Planning System (Varian Medical Systems, Palo Alto, USA) to achieve flash in VMAT plans.^9^, ^10^ In these methods, ∼1 cm of bolus is applied to the skin in the region of the planning target volume (PTV), and the PTV is expanded into this region. The treatment plan is optimized to this expanded PTV, forcing multi‐leaf collimator (MLC) leaves to open beyond the PTV, which creates a flash region. A final dose calculation is performed with the bolus removed, and the dose to the original, non‐expanded PTV is assessed. In clinical practice, multiple iterative rounds of optimizing, recalculating, and renormalizing are required to generate an acceptable plan. Treating the regional lymph nodes further complicates the planning process, as the bolus is typically only added in the breast or chestwall region.

A new optimization and treatment delivery technique, RapidArc dynamic (RAD), has recently been released in Eclipse Treatment Planning System (Varian Medical Systems, Palo Alto, USA). RAD integrates the benefits of static‐gantry IMRT and VMAT by allowing the user to add static‐gantry modulated ports within a VMAT arc. The optimization creates an arc‐based treatment plan with integrated static‐gantry angle ports with fluence modulation and also has the option for dynamic collimator rotation throughout arc delivery. The new optimizer allows the user to add automatic skin flash (ASF) to RAD delivery, obviating the manual addition of virtual bolus or pseudo flash. The optimizer automatically applies a virtual bolus for optimization and removes the bolus for calculation, automating the multi‐step virtual bolus process. The user specifies the thickness and Hounsfield unit (HU) of the bolus for the ASF. However, the ideal thickness and HU of this flash region is not known for RAD.^11^

In this work, we aim to determine, in the context of breast and chestwall treatment, whether the ASF feature generates high‐quality plans that are robust to simulated setup uncertainty. We also aim to provide the community with recommended HU and flash region thickness to be used in the ASF tool. The ideal ASF HU and thickness will balance plan quality and plan robustness to shifts. We evaluate target and OAR dosimetry and plan robustness to patient motion by optimizing with varying ASF HU and thickness.

## 2 METHODS

### 2.1 RapidArc dynamic optimization

RAD combines VMAT arc delivery with static gantry angle modulated ports. The user selects the arc start angle, arc end angle, modulated port angles, and the relative weighting between arc and port control points. The relative weighting between arcs and ports ranges from −2 to +2, with the following definitions: −2 (Arc Dominant), −1 (Arc), 0 (Balanced), 1 (Static), or 2 (Static Dominant). 0 (Balanced) was selected for all plans. Further, the user has three options in selecting dynamic collimator options: (1) The user can specify one fixed collimator angle for each arc (similar to traditional VMAT). (2) The user can specify the collimator angle for the start and end of each arc and for each modulated port, allowing the optimizer to dynamically adjust the collimator angle in between these points. (3) The optimizer fully and dynamically optimizes the collimator at all points, including during the arc and modulated ports. In the optimizer, the user must specify the maximum number of optimization iterations (possible range: 100–4000, default = 800); 1000 was selected for all plans.

**FIGURE 1:**
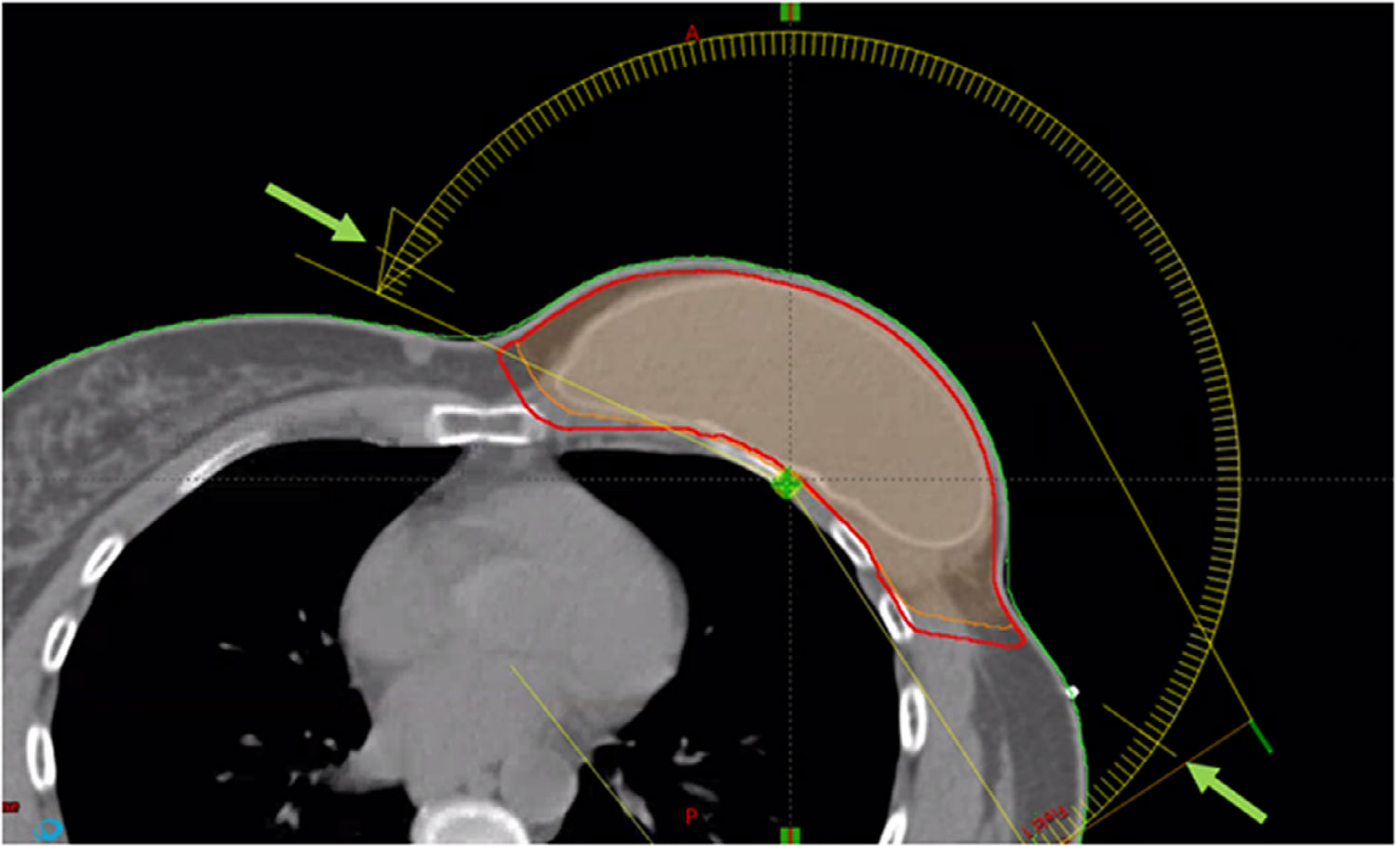
The isocenter is placed at the lung/chestwall interface. The modulated port angles are indicated by the green arrows. The CTV is shown in orange and PTV in red. CTV, clinical target volume; PTV, planning target volume.

For 13 whole breast or chestwall patients without regional lymph nodes (9 left‐sided and 4 right‐sided), treatment plans were created with a single VMAT arc spanning between 200° and 220° and two modulated ports. All patients were simulated in deep inspiration breath hold (DIBH) using the SDX spirometry‐based respiratory gating system (DYN'R Medical Systems). The isocenter was placed at or near the chestwall/lung interface in the plane near mid‐target in the superior‐inferior direction. The arc and modulated port angles selected were based on patient‐specific anatomy. The medial arc start angle was at least 1 cm anterior to the contralateral breast to avoid the beam entering through it, approximately where a medial tangent would be placed. The lateral arc border extended 20°–30° beyond a typical lateral tangent angle. Each plan also used two static angle modulated ports placed approximately where conventional tangent fields would be placed. The first modulated port was selected using the beam's eye view to limit the heart and lung in the field while irradiating the maximum amount of breast or chestwall target. The second modulated port was placed 160°–176° from the first port. Modulated ports were not directly opposed to allow additional degrees of freedom in the optimization. Treatment field geometry and patient characteristics are shown in Table 1. The field aperture was limited to 20 cm in the x‐jaw direction to limit the amount of MLC over‐ranging in the plan. Figure 1 demonstrates an example of isocenter placement and arc geometry. The collimator angle was selected at the beginning and end of each arc and at each static‐angle modulated port to align with the angle of the breast and lung interface, allowing the optimizer to dynamically change the angle between modulated ports.

**TABLE 1:** Patient target characteristics and treatment field geometry.

Patient	Laterality	Target type	CTV volume (cc)	PTV volume (cc)	Arc start angle (°)	Arc end angle (°)	Arc length (°)	Medial port angle (°)	Lateral port angle (°)
**1**	left	CW‐ no recon	145	216	300	150	210	311	135
**2**	left	CW‐ no recon	116	208	290	150	220	290	130
**3**	left	flap recon	563	619	290	150	220	295	130
**4**	left	flap recon	804	924	300	150	210	300	125
**5**	left	implant recon	618	705	300	150	210	300	125
**6**	left	intact breast	804	986	310	150	200	315	145
**7**	left	intact breast	644	757	300	150	210	300	125
**8**	left	intact breast	659	785	300	150	210	300	125
**9**	left	intact breast	211	271	300	150	210	305	140
**10**	right	CW‐ no recon	429	542	60	210	210	55	231
**11**	right	CW‐ no recon	215	339	60	210	210	55	223
**12**	right	implant recon	813	925	65	210	215	60	230
**13**	right	intact breast	592	716	60	210	210	60	230

Abbreviations: CW, chestwall; CTV, clinical target volume; PTV, planning target volume; RECON, reconstruction.

The external patient contour was generated using the “Search Body” contouring tool with a lower threshold set to −350 HU. Clinical target volumes (CTVs) were drawn by a radiation oncologist according to consensus guidelines.^12^, ^13^ The PTV was generated by expanding the CTV by 5 mm and then cropping any portions that extend into the chestwall muscle and cropping 3 mm from the skin. The resulting PTV overlaps with the CTV on the anterior surface (Figure 1).

The dose prescription was 40.05 Gy in 15 fractions, and 6 MV was used in all plans. The planning goals are outlined in Table 2. All plans were normalized such that the PTV D95% = 95%.

**TABLE 2:** Treatment planning goals.

Structure	Objective	Variation	Priority
PTV	D95% ≥ 38.05 Gy	≥ 36.05 Gy	1
PTV	V36.05Gy ≥ 99%	≥ 98%	1
PTV	V105% ≤10%	≤ 15%	1
Heart (left‐sided)	*D*_mean_ < 2 Gy	< 5 Gy	2
Heart (right‐sided)	*D*_mean_ < 1 Gy	< 5 Gy	2
Ipsilateral lung	*D*_mean_ < 7 Gy	< 13 Gy	2
Ipsilateral lung	V16Gy ≤ 15%	≤ 20%	2
Ipsilateral lung	V4Gy ≤ 35%	≤ 50%	2
Contralateral lung	*D*_mean_ < 1 Gy	< 2.5 Gy	3
Contralateral lung	V4Gy ≤ 10%	≤ 15%	3
Contralateral breast	*D*_mean_ < 1 Gy	< 3 Gy	4

Abbreviations: PTV, planning target volume.

The ASF tool in the optimizer can be used to optimize with skin flash. The user selects the structure on which the flash is added, the thickness of the flash region (0 to 40 mm), and the HU of the flash region (−500 to 500 HU). The ASF thickness can be symmetrical or asymmetrical. The default HU is the median of the selected target, and there is no default thickness. A RAD plan was created without any skin flash. The same plan was then copied and re‐optimized with varying HU and thickness in the ASF. With 0 and −350 HU, ASF thicknesses of 0, 5–10, 15, and 20 mm were used to determine an optimal thickness. The optimal HU value was determined by using a single thickness of 7 mm and reoptimizing with HU values of −500 and −200. The RAD optimization window does not allow real‐time user interaction or adjustments to optimization objectives and does not have the option to continue from a previous optimization; all re‐optimizations were from the beginning of the optimization process, but optimization objectives were not changed. A conventional RapidArc plan without any added skin flash was also created for comparison.

### 2.2 Plan quality analysis

To assess the plan quality, the DVH metrics in Table 2 were recorded in each plan created with varying HU and thickness of the ASF. Using an ASF HU of 0 and −350 HU, the flash thickness was varied from 0–20 mm. Two‐tailed paired *t*‐tests were used to determine if the amount of flash thickness made a significant (*p* ≤ 0.05) difference, using the 7 mm of ASF plan as a baseline. For 7 mm of flash, the HU was varied from 0 to −500 HU, and the DVH metrics were compared to those in the 0 HU plan using a two‐tailed paired *t*‐test (*p* ≤ 0.05).

### 2.3 Robustness analysis

**FIGURE 2:**
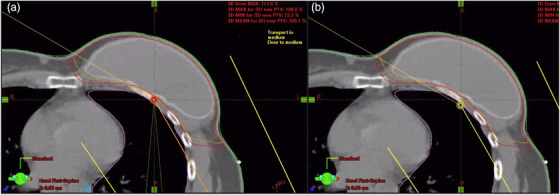
(a) The original isocenter location in indicated by the orange circle in the chestwall. (b) the isocenter is shifted 5 mm posteriorly and is indicated by the yellow circle at the lung/chestwall interface.

To assess plan robustness, the isocenter was shifted 5 mm posteriorly in all plans (Figure 2), moving the breast or chestwall anteriorly into the flash region. 5 mm was selected for this study, as this cohort of patients was treated using DIBH and with daily cone‐beam computed tomography (CBCT) imaging. The CTV D95%, and D99% were compared between the shifted and non‐shifted plans and change in coverage was reported. A two‐tailed paired *t*‐test was used to assess whether the change in CTV coverage was significant (*p* ≤ 0.05) between plans with varying HU and thickness of ASF, and between plans with ASF compared to no ASF.

## 3 RESULTS

### 3.1 Plan quality analysis–effect of automatic skin flash (ASF) thickness

DVH metrics were recorded for each plan. The mean ± standard deviation for each DVH metric for the 13 patients is recorded in Tables 3 (0 HU) and 4 (−350 HU). Highlighted values indicate metrics that were significantly different than those in the plan with 7 mm of flash. For the 0 HU plans, no metrics were significantly different for 5, 6, 8, 9, or 10 mm of skin flash than in the 7 mm plan. Some significant differences in plan quality exist when comparing the 7 mm plan to the plans with no flash or with 15, 20 mm of flash. The only DVH metric that was significantly different and outside of clinical limits was the PTV V105% in the 15 mm and 20 mm plans; all other dose metrics were within clinically acceptable limits. For the −350 HU plans, significant differences were mainly in the ipsilateral lung and PTV V36.05Gy but all differences were within clinically acceptable limits.

**TABLE 3:** Planning metrics for plans created with varying ASF thickness with 0 HU.^a^

ASF thickness (mm)	0 (RA)	0 (RAD)	5	6	7	8	9	10	15	20
HU			−350	−350	−350	−350	−350	−350	−350	−350
PTV D95% (%)	38.1	38.1	38.1	38.1	38.1	38.1	38.1	38.1	38.1	38.1
PTV V36.05Gy (%)	99.4 ± 0.3	99.6 ± 0.2	99.5 ± 0.3	**99.6 ± 0.2**	99.5 ± 0.4	99.5 ± 0.3	99.4 ± 0.3	99.4 ± 0.3	**99.2 ± 0.4**	**99.1 ± 0.3**
PTV V105% (%)	0.7 ± 1.1	0.0 ± 0.0	1.8 ± 2.6	1.6 ± 2.4	3.7 ± 7.3	2.7 ± 4.7	4.7 ± 6.0	4.6 ± 5.8	**11.3 ± 11.6**	**16.9 ± 12.8**
Heart *D*_mean_ (Gy)	**1.2 ± 0.4**	**1.1 ± 0.3**	1.3 ± 0.5	1.3 ± 0.4	1.3 ± 0.4	1.3 ± 0.4	1.3 ± 0.4	1.3 ± 0.5	1.4 ± 0.5	1.4 ± 0.6
Ipsilateral lung *D*_mean_ (Gy)	5.8 ± 1.0	**5.2 ± 0.9**	5.7 ± 0.9	5.7 ± 0.9	5.8 ± 1.1	5.8 ± 1.1	5.8 ± 1.1	5.8 ± 1.1	6.0 ± 1.2	**6.3 ± 1.2**
Ipsilateral lung V16 Gy (%)	12.1 ± 3.0	**11.3 ± 2.9**	**12.6 ± 3.2**	12.6 ± 3.4	12.8 ± 3.5	12.8 ± 3.4	13.0 ± 3.7	13.1 ± 3.7	**13.5 ± 3.9**	**13.9 ± 4.0**
Ipsilateral lung V4Gy (%)	31.8 ± 5.3	**27.6 ± 5.1**	29.5 ± 4.6	29.8 ± 4.6	30.10 ± 5.9	29.7 ± 5.6	29.7 ± 5.3	29.7 ± 5.4	30.3 ± 5.2	31.7 ± 5.9
Contralateral lung *D*_mean_ (Gy)	0.9 ± 0.2	**0.6 ± 0.2**	0.7 ± 0.3	0.7 ± 0.3	0.8 ± 0.3	0.7 ± 0.3	0.7 ± 0.3	0.7 ± 0.3	0.8 ± 0.3	0.7 ± 0.3
Contralateral lung V4Gy (%)	1.0 ± 0.9	**0.4 ± 0.6**	0.6 ± 0.8	0.7 ± 0.9	0.8 ± 1.1	0.8 ± 1.0	0.7 ± 1.0	0.6 ± 0.8	1.0 ± 2.1	0.5 ± 0.8
Contralateral breast *D*_mean_ (Gy)	1.2 ± 0.3	0.9 ± 0.4	1.0 ± 0.4	1.1 ± 0.3	0.9 ± 0.3	1.0 ± 0.4	1.0 ± 0.4	1.0 ± 0.4	1.0 ± 0.4	**1.1 ± 0.4**

^a^ Bolded values indicate statistically significant difference compared to the same metric for the plan with 7 mm of ASF (*p* ≤ 0.05).

Abbreviations: ASF, automatic skin flash; HU, Hounsfield unit; PTV, planning target volume; RA, RapidArc; RAD, RapidArc dynamic.

**TABLE 4:** Planning metrics for plans created with varying ASF thickness with −350 HU.^a^

ASF thickness (mm)	0 (RA)	0 (RAD)	5	6	7	8	9	10	15	20
HU			0	0	0	0	0	0	0	0
PTV D95% (%)	38.1	38.1	38.1	38.1	38.1	38.1	38.1	38.1	38.1	38.1
PTV V36.05Gy (%)	99.4 ± 0.3	99.6 ± 0.2	99.5 ± 0.3	99.7 ± 0.3	99.6 ± 0.3	99.6 ± 0.4	**99.5 ± 0.3**	**99.5 ± 0.3**	**99.3 ± 0.4**	**99.2 ± 0.4**
PTV V105% (%)	0.7 ± 1.1	0.0 ± 0.0	2.2 ± 2.8	2.43± 2.3	1.5 ± 2.8	1.7 ± 3.3	2.1 ± 3.6	2.3 ± 4.0	5.5 ± 8.4	**6.7 ± 8.5**
Heart *D*_mean_ (Gy)	**1.2 ± 0.4**	**1.1 ± 0.3**	1.3 ± 0.5	1.3 ± 0.4	1.3 ± 0.4	1.3 ± 0.4	1.3 ± 0.4	1.4 ± 0.4	1.4 ± 0.5	1.5 ± 0.5
Ipsilateral lung *D*_mean_ (Gy)	5.8 ± 1.0	**5.2 ± 0.9**	5.6 ± 0.9	5.7 ± 0.9	5.8 ± 1.0	**5.8 ± 1.0**	**5.9 ± 1.1**	**5.9 ± 1.1**	**6.1 ± 1.2**	**6.4 ± 1.3**
Ipsilateral lung V16Gy (%)	12.1 ± 3.0	**11.3 ± 2.9**	**12.2 ± 3.3**	12.5 ± 3.3	12.7 ± 3.5	**12.8 ± 3.4**	**13.1 ± 3.8**	**13.1 ± 3.8**	**13.7 ± 4.0**	**14.2 ± 4.0**
Ipsilateral lung V4Gy (%)	31.8 ± 5.3	**27.6 ± 5.0**	29.7 ± 4.6	29.9 ± 4.4	30.2 ± 5.4	30.0 ± 5.5	30.0 ± 5.1	30.0 ± 5.0	30.9 ± 5.4	**32.6 ± 6.3**
Contralateral lung *D*_mean_ (Gy)	0.9 ± 0.2	**0.6 ± 0.2**	0.7 ± 0.3	0.7 ± 0.3	0.8 ± 0.4	0.8 ± 0.4	0.8 ± 0.3	0.7 ± 0.3	0.8 ± 0.4	0.8 ± 0.3
Contralateral lung V4Gy (%)	1.0 ± 0.9	**0.4 ± 0.6**	0.7 ± 0.9	0.7 ± 1.0	1.1 ± 1.3	1.0 ± 1.3	0.8 ± 1.4	0.9 ± 1.1	1.5 ± 2.3	0.8 ± 0.8
Contralateral breast *D*_mean_ (Gy)	1.2 ± 0.3	**0.9 ± 0.4**	1.0 ± 0.4	1.1 ± 0.3	1.1 ± 0.4	1.0 ± 0.4	1.0 ± 0.4	1.0 ± 0.4	1.1 ± 0.4	1.1 ± 0.4

^a^ Bolded values indicate statistically significant difference compared to the same metric for the plan with 7 mm of ASF (*p* ≤ 0.05).

Abbreviations: ASF, automatic skin flash; HU, Hounsfield unit; PTV, planning target volume; RA, RapidArc; RAD, RapidArc dynamic.

**FIGURE 3:**
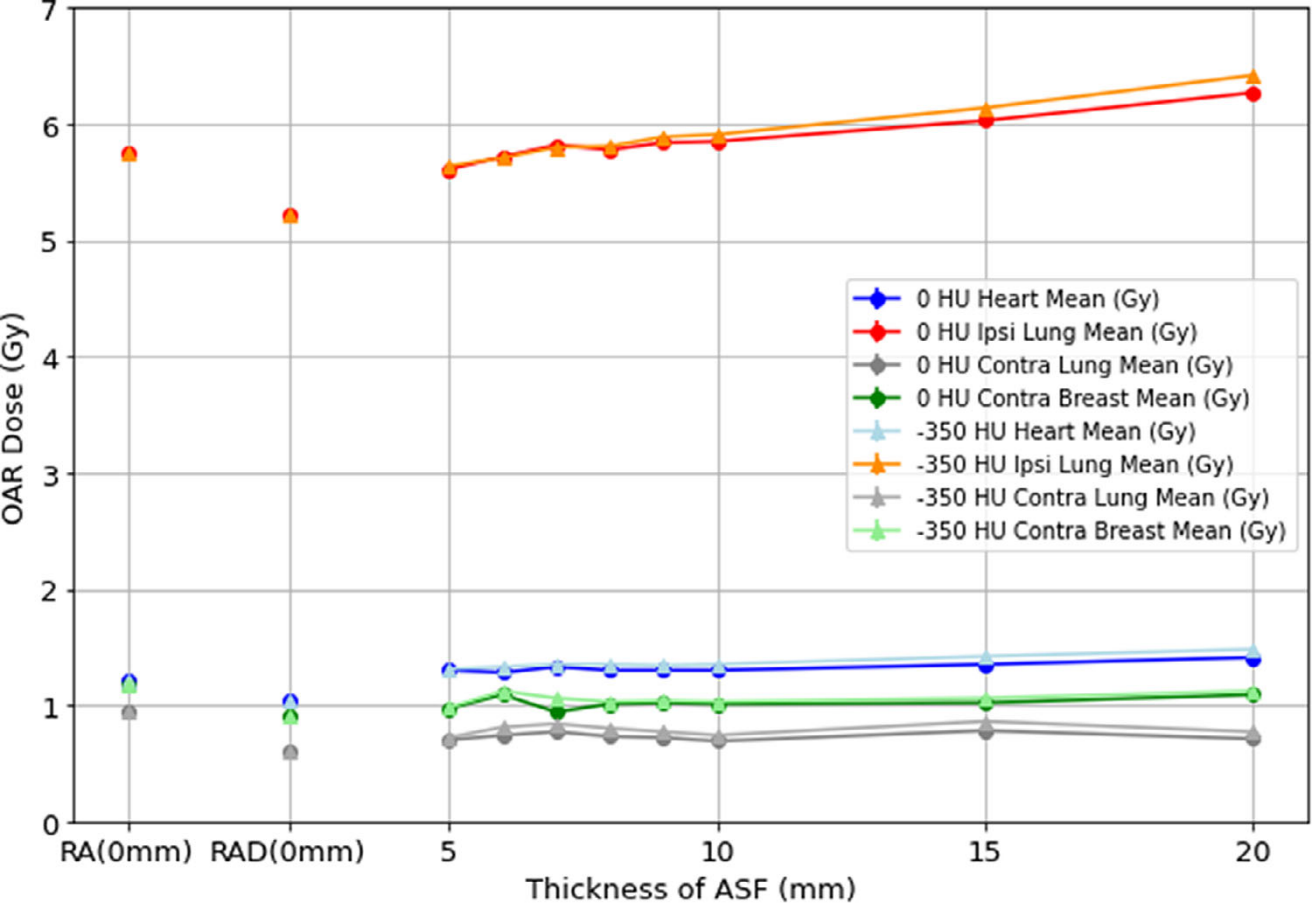
Mean doses for heart, ipsilateral and contralateral lungs, and contralateral breast for plans created with no ASF, and 5–20 mm of ASF with 0 and −350 HU. ASF, automatic skin flash. HU, Hounsfield unit.

The above data is shown in Figures 3 (DVH metrics reported in Gy) and 4 (DVH metrics reported in %). Ipsilateral lung *D*_mean_, V16Gy, and V4Gy, Heart *D*_mean_, and PTV V105% generally increased with increasing ASF thickness. The remaining DVH metrics did not demonstrate a trend related to ASF thickness. The results for 0 and −350 HU were similar except for the PTV V105%. The increase in V105% was markedly more pronounced in the 0 HU plans than in the −350 HU plans. The PTV V105% was higher in any plans with ASF added than in the RA or RAD plans without flash but within clinically acceptable limits for thicknesses ≤ 10 mm.

**FIGURE 4:**
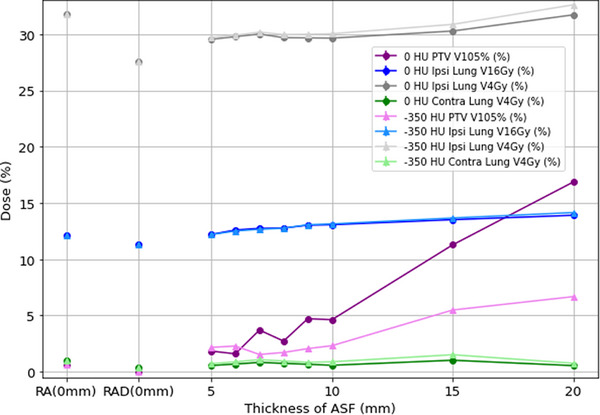
Ipsilateral lung V16Gy and V4Gy, contralateral lung V4Gy, and contralateral breast PTV V105% for plans created with no ASF, and 5–20 mm of ASF with 0 and −350 HU. ASF, automatic skin flash; HU, Hounsfield unit; PTV, planning target volume.

### 3.2 Plan quality analysis–effect of ASF Hounsfield unit (HU)

Table 5 records the mean ± standard deviation for the 13 patients for each HU assignment. A conventional RA plan and a RAD plan with no added flash are included for comparison. Bolded values indicate metrics that were significantly different from those in the 0 HU RAD plan.

**TABLE 5:** Planning metrics for 7 mm ASF thickness, and HU assignments of 0 to −500 HU.^a^

ASF thickness (mm)	0 (RA)	0 (RAD)	7	7	7	7
HU			0	−200	−350	−500
PTV D95% (%)	38.1	38.1	38.1	38.1	38.1	38.1
PTV V36.05Gy (%)	99.4 ± 0.3	99.6 ± 0.2	99.5 ± 0.4	99.5 ± 0.4	**99.6 ± 0.3**	**99.6 ± 0.4**
PTV V105% (%)	0.7 ± 1.1	0.0 ± 0.0	3.7 ± 7.3	2.7 ± 5.9	1.5 ± 2.8	2.7 ± 4.5
Heart *D*_mean_ (Gy)	1.2 ± 0.4	1.1 ± 0.3	1.3 ± 0.4	**1.4 ± 0.4**	1.3 ± 0.4	1.4 ± 0.4
Ipsilateral lung *D*_mean_ (Gy)	5.8 ± 1.0	5.2 ± 0.9	5.8 ± 1.1	**5.9 ± 1.1**	5.8 ± 1.0	5.8 ± 1.0
Ipsilateral lung V16Gy (%)	12.1 ± 3.0	11.3 ± 2.9	12.8 ± 3.5	12.8 ± 3.5	12.7 ± 3.4	12.6 ± 3.4
Ipsilateral lung V4Gy (%)	31.8 ± 5.3	27.6 ± 5.1	30.0 ± 5.9	**30.3 ± 5.8**	30.2 ± 5.4	30.5 ± 5.3
Contralateral lung *D*_mean_ (Gy)	0.9 ± 0.2	0.6 ± 0.2	0.8 ± 0.3	**0.8 ± 0.3**	**0.8 ± 0.4**	**0.9 ± 0.4**
Contralateral lung V4Gy (%)	1.0 ± 0.9	0.4 ± 0.6	0.8 ± 1.1	0.9 ± 1.2	1.1 ± 1.4	**1.2 ± 1.4**
Contralateral breast *D*_mean_ (Gy)	1.2 ± 0.3	0.9 ± 0.4	0.9 ± 0.3	1.0 ± 0.3	1.1 ± 0.4	**1.1 ± 0.4**

^a^ Bolded values indicate metrics that were significantly different from those in the 0 HU RAD plan (*p* ≤ 0.05).

Abbreviations: ASF, automatic skin flash; HU, Hounsfield unit; PTV, planning target volume; RA, RapidArc; RAD, RapidArc dynamic.

The PTV V105% increased with 0 HU assigned to the ASF region but was not significantly different from other HU assignments and was within clinically acceptable limits. All other DVH metrics were relatively unchanged, indicating that with 7 mm of ASF, the plan quality was not affected by HU selection.

### 3.3 Robustness analysis

**FIGURE 5:**
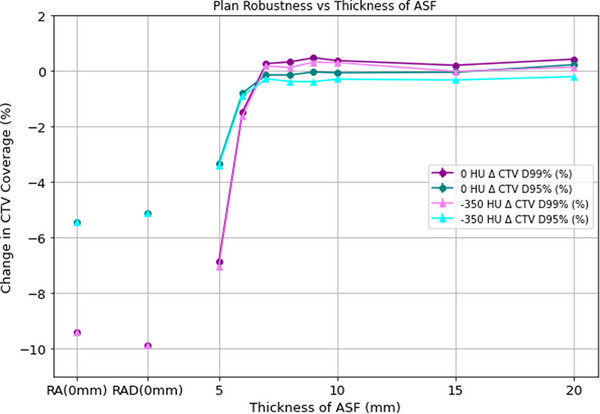
The change in CTV D95% and D99% when a 5 mm isocenter shift is introduced are shown for plans with 0 and −350 HU and varying flash margin thickness. CTV, clinical target volume.

Robustness was assessed by introducing a 5 mm posterior isocenter shift and measuring the change in CTV D95% and D99% metrics for all plans. Results for the 0 and −350 HU plans are shown in Figure 5. For both HU values, RA plans and RAD plans with at least 6 mm of flash were significantly more robust than RA and RAD plans created with no flash. In the 6 mm plans, there was some decrease in CTV coverage, though not statistically significant. Plans with 7 mm or more flash were robust to 5 mm shifts, and increasing the flash margin beyond 7 mm did not increase robustness.

The change in CTV coverage metrics for plans with a 7 mm flash region of varying HU values from −500 to 0 HU was also assessed. Any HU (from 0 to −500 HU) resulted in plans robust to 5 mm isocenter shifts; there was no change in robustness with changing HU.

## 4 DISCUSSION

Applying skin flash using the ASF tool generated treatment plans meeting planning objectives. Increasing the flash region thickness in the ASF tool generally resulted in small increases to OAR doses. The only metric with a large increase was the PTV V105% in plans with > 10 mm of flash. The increase in V105% was much more pronounced when 0 HU was used in the flash region compared to when −350 HU was used. The plans created with 0 and −350 HU were found to be similarly robust to isocenter shift. Thus, −350 HU is recommended over 0 HU in the flash region to limit the PTV V105%. There were no statistically significant differences in the PTV V105% between the −350 HU plans and the −200 or −500 HU plans. Thus, we recommend −350 HU as a starting point, and the HU can be adjusted for each patient plan to obtain optimal results. For 5 mm isocenter shifts, we found that 7 mm of flash provided sufficient robustness against 5 mm isocenter shifts for a CTV that is cropped 3 mm from the patient surface. There was no gain in robustness by adding flash beyond 7 mm.

Lizondo et al. performed a similar analysis on the dosimetric quality and robustness of VMAT plans generated using virtual bolus techniques.^11^ They applied a virtual bolus of 5 or 10 mm thickness and tested HU values ranging from −700 to 0 HU. They conclude that the optimal virtual bolus thickness to account for a 5 mm shift is equal to the CTV‐PTV margin plus 5 mm, and an HU around −500 to −400 should be used. This aligns well with our results. In their work, the CTV extends to the patient surface, and the “PTV” refers to the PTV that has been expanded into the virtual bolus region. So in their case, the CTV‐PTV margin is 5 mm, for a recommended total of 10 mm of flash. Our results showed similar findings in that 7–10 mm and −350 HU of flash were optimal.

In this work, plan robustness was only evaluated against 5 mm posterior isocenter shifts. In reality, the interfraction variability for a breast or chestwall can be much greater than 5 mm, and the breast position and shape will change in the medial‐lateral and superior‐inferior directions as well. An isocenter shift in the posterior‐only direction was selected to represent anterior respiratory motion, typically the direction with the largest magnitude of respiratory motion.^14^, ^15^, ^16^, ^17^ Further, since the ASF tool applied flash in 3 dimensions, a 5 mm shift in one cardinal direction is a larger effective shift than 5 mm split over 3 dimensions.The amount of flash used in RAD planning must take into account setup uncertainties, image guidance type and frequency in each clinic, and respiratory motion management, as well as patient‐specific factors. A 5 mm shift is reasonable given daily image guidance and the use of a reliable DIBH method, but would not be applicable with less frequent imaging or if respiratory motion is present. For breast positional changes > 5 mm, a larger ASF thickness should be evaluated. In selecting between 7–10 mm of flash, the planner must balance the amount of flash required for the clinical scenario and the increase in PTV V105%. Using a lower (–350 HU) assignment in the flash region was found to mitigate some of the PTV V105% increase. The mean PTV V105% in plans with 20 mm and −350 HU was 6.4% but ranged from 0.5% to 25.8%. For some patients, flash thickness can be increased up to 20 mm without adversely affecting plan quality.

With thicker and higher HU flash regions, the PTV V105% generally increased. For those plans with large PTV V105%, the optimizer showed that the PTV V105% was 0 or close to 0. After the final dose calculation, however, the PTV V105% was much higher given the same PTV D95% coverage. This change in target dose homogeneity was evident in the DVH shape after the final dose calculation. Decreasing the PTV V105% in the optimizer did not decrease it in the final dose calculation. The discordance is due to the intermediate dose calculation being performed with a “virtual bolus” in place and the final dose calculation without. The effect could be reduced by decreasing the HU or the thickness in the ASF tool.

This study evaluated a relatively small number of patients and did not include any treatment planning for patients with regional nodal irradiation (RNI). Regional lymph node treatment was intentionally excluded as flash is not used on nodal target volumes. The ASF tool allows the user to select which target structure(s) to apply flash; in cases with RNI, separate optimization structures will be required to apply the ASF tool.

This study evaluated the ASF tool with only one option regarding the dynamic collimator, the arc and modulated port geometry, and relative weighting. Assessment of the ASF tool under different conditions is warranted before clinical use.

The ASF tool was not directly compared to conventional virtual bolus methods in this work. We therefore cannot conclude that our plans were equivalent or better in plan quality or robustness to plans created using virtual bolus methods. The majority of DVH metrics in plans created using ASF met the criteria for clinical acceptability, and with the exception of the PTV V105% and V36.05Gy, most metrics were equivalent or lower than those in the RapidArc plan (which did not use a virtual bolus). Using the ASF tool was less time consuming and required less iterations than planning using a virtual bolus based on the authors’ clinical experiences.

## 5 CONCLUSION

With the thickness and HU parameters tested, the ASF tool produced high‐quality treatment plans meeting DVH objectives. Using the ASF tool automated a conventionally iterative process. There was little to no difference in plan quality or plan robustness against 5 mm shifts in plans created with 7–10 mm of skin flash. The PTV V105% increased with ASF thickness and this increase was much larger in plans with 0 HU compared to those with −350 HU. For plans with 7 mm of flash ranging from 0 to −500 HU, there was no change in robustness. We therefore recommend using the ASF tool in breast and chestwall RAD plans with an HU of −350 and with a thickness of 7–10 mm when assuming 5 mm of interfraction motion. The exact thickness should balance limiting the PTV V105% with the required robustness depending on IGRT and patient factors.

## AUTHOR CONTRIBUTIONS

All of the above listed authors have contributed directly to the intellectual content, preparation, and final review of this manuscript.

## CONFLICT OF INTEREST STATEMENT

Emily Hubley has received honoraria from Varian Medical Systems for speaking about RapidArc Dynamic. This work was partially funded by Varian Medical Systems.
